# Pre-operative High-Resolution CT and MRI Evaluation in Pediatric Cochlear Implant Candidates: Correlation With Surgical Findings and Outcomes

**DOI:** 10.7759/cureus.103441

**Published:** 2026-02-11

**Authors:** Namrata Mehta, Viral Patel, Urvi Amin, Jayesh Bhatt, Girish Mishra, Mehul Patel, Nilay Shah, Riddhi Gohil

**Affiliations:** 1 Department of Radiodiagnosis, Pramukhswami Medical College and Shree Krishna Hospital, Bhaikaka University, Karamsad, Anand, IND; 2 Department of Otolaryngology, Head and Neck Surgery, Pramukhswami Medical College and Shree Krishna Hospital, Bhaikaka University, Karamsad, Anand, IND; 3 Department of Otolaryngology, Head and Neck Surgery, IRIS Hospital, Anand, IND

**Keywords:** cochlear implantation, cochlear nerve, high-resolution ct, inner ear malformations, mri temporal bone, pediatric sensorineural hearing loss

## Abstract

Purpose

Congenital sensorineural hearing loss (SNHL) is a major childhood disability, and early cochlear implantation offers optimal auditory and language outcomes. High-resolution computed tomography (HRCT) and magnetic resonance imaging (MRI) are essential in evaluating candidacy, identifying inner ear malformations (IEMs), assessing cochlear nerve integrity, and predicting surgical challenges. This study evaluates the role of HRCT and MRI in pediatric cochlear implant candidates and correlates imaging findings with intraoperative events and postoperative outcomes.

Methods

This retrospective study included 32 children (<7 years) with congenital SNHL who underwent HRCT and MRI of the temporal bones. HRCT assessed mastoid pneumatization, middle ear status, vascular variants, facial recess anatomy, and cochlear morphology. MRI evaluated the membranous labyrinth, cochleovestibular nerve, internal auditory canal (IAC), and brain. Exclusion criteria included lack of consent, age >7 years, metallic implants, pacemakers, or prior cochlear implantation. Surgical records were retrospectively reviewed to assess operative complexity, including duration of surgery, intraoperative challenges, and complications encountered.

Results

Among 32 patients (mean age 2.9 years), mastoid aeration was normal in 81.25%, and the middle ear cavity was aerated in 90.63%. Sigmoid sinus variants, high-riding jugular bulbs (18.75%), and low-lying dural plates (34.38%) were common. Vestibular aqueduct anomalies were identified in 18.75%. The cochlear aperture was normal in 74.88%, widened in 12.5%, and stenosed in 6.25%. Inner ear malformations were present in 15.63%, including large vestibular aqueduct syndrome (LVAS), incomplete partitions, and cochlear hypoplasia, with cochlear nerve aplasia in two cases. Nineteen patients underwent cochlear implantation; all IEM cases experienced intraoperative perilymphatic gushers, consistent with imaging predictions. All surgeries used the transmastoid facial recess approach with an extended round window technique. Postoperative recovery was uneventful in all cases.

Conclusions

HRCT and MRI together provide a comprehensive assessment of pediatric cochlear implant candidates, enabling precise identification of IEMs, cochlear nerve anomalies, and surgical risk factors. Imaging reliably predicts intraoperative challenges such as gushers and aids in tailoring surgical approaches. A combined CT-MRI protocol is indispensable for optimizing safety, minimizing complications, and enhancing postoperative outcomes in pediatric cochlear implantation.

## Introduction

Congenital sensorineural hearing loss (SNHL) is one of the most prevalent birth disorders, which occurs in about one out of every thousand live births [1]. The World Health Organization (WHO) estimates that 34 million children have hearing loss requiring rehabilitation, and the numbers are increasing [2]. It occurs due to aberrations that affect the hair cells localized in the membranous labyrinth, anomalies in the vestibulocochlear nerve and the inner ear, as well as due to conditions that affect the brain's auditory processing centers.

Over the past few decades, cochlear implant (CI) surgery has been on a rising trend and has transformed the treatment of severe to profound sensorineural hearing loss (SNHL) in both adults and children.

Cochlear implants (CIs) are the treatment of choice for severe to profound sensorineural hearing loss in patients who are refractory to conventional hearing augmentation [3].

Imaging plays a critical role in the evaluation of various causes of deafness and, more importantly, aids in identifying potential candidates who would benefit from cochlear implant. It not only identifies inner ear congenital and acquired abnormalities or cochlear nerve anomalies but also detects various temporal bone abnormalities that can pose difficulties during surgery [3,4].

Magnetic resonance imaging (MRI) and/or high-resolution computed tomography (HRCT) are the main imaging methods used to evaluate the temporal bone. MRI has a complementary role to CT, especially for patients with a narrow internal auditory canal (IAC) and suspected cochlear nerve aplasia due to an inner ear deformity [5,6]. CT offers better spatial resolution compared to MRI and has the advantage of providing outstanding topographic details, devoid of superimposition from structures and is less prone to artifacts. In addition to providing anatomical information that is crucial for surgical planning, preoperative high-resolution CT of the temporal bone enables the further detection of middle and external ear disorders [7].

Contrarily, MRI provides excellent soft-tissue resolution as compared to CT and has the additional advantage of no exposure to ionizing radiation [3]. It serves as a valuable tool in the preoperative assessment of cochlear implant candidates, as it simultaneously evaluates the inner ear, cochleovestibular nerve, cerebellopontine angle, and brain to exclude associated central or peripheral causes of hearing loss [8].

The study aims to systematically analyze HRCT and MRI findings in patients with sensorineural hearing loss, compare their respective roles in cochlear implant evaluation, and determine how imaging findings impact implant candidacy and preoperative surgical planning.

## Materials and methods

This retrospective study was conducted in the Department of Radiodiagnosis, Shree Krishna Hospital, Karamsad, after approval from the Institutional Ethics Committee (IEC/BU/150/Faculty/10/359/2023). Previously acquired imaging data of pediatric patients were reviewed. Multi-detector computed tomography (MDCT) was performed on a GE Optima CT660 (128-slice) scanner (manufactured by GE Healthcare, Bangalore, India) under sedation or short-acting general anesthesia for children under seven years. Non-contrast high-resolution CT scans of the temporal bones were obtained and reformatted into thin contiguous coronal and sagittal sections. MRI was performed using a Siemens Magnetom Spectra 3T scanner (manufactured by Siemens Healthineers, Bangalore, India).

Non-contrast MRI of both temporal bones was performed using 0.7 mm 3D-constructive interference in steady state (CISS heavily T2W) and 0.7 mm 3D-volumetric interpolated breath-hold examination (VIBE T1W) axial sequences, 2 mm T1W and T2W coronal images, 1 mm T2Space sagittal, and 2 mm T2W sagittal images. CISS (heavily T2W) reconstructions were then obtained perpendicular to the internal auditory canals. Non-contrast MRI screening of the brain was performed by obtaining 4 mm-thick T1W, PD-T2, diffusion-weighted and apparent diffusion coefficient (ADC) axial images. Surgical records were retrospectively reviewed to assess operative complexity, including duration of surgery, intraoperative challenges, and surgical complications encountered.

Inclusion criteria

Paediatric patients under seven years of age with congenital sensorineural hearing loss are referred for CT and MRI of the temporal bones (as brain plasticity for hearing and speech development is optimal below this age).

Exclusion criteria

Patients above seven years, those without parental or guardian consent, and children with pacemakers, metallic implants, or existing cochlear implants.

## Results

A total of 32 patients were included in the study, with ages ranging from birth to four years (mean age: 2.9 years). The majority were between two and three years (40.6%), followed by one to two years (28.1%). Males predominated, with a male-to-female ratio of 2.2:1.

Mastoid and middle ear findings

**Figure 1:**
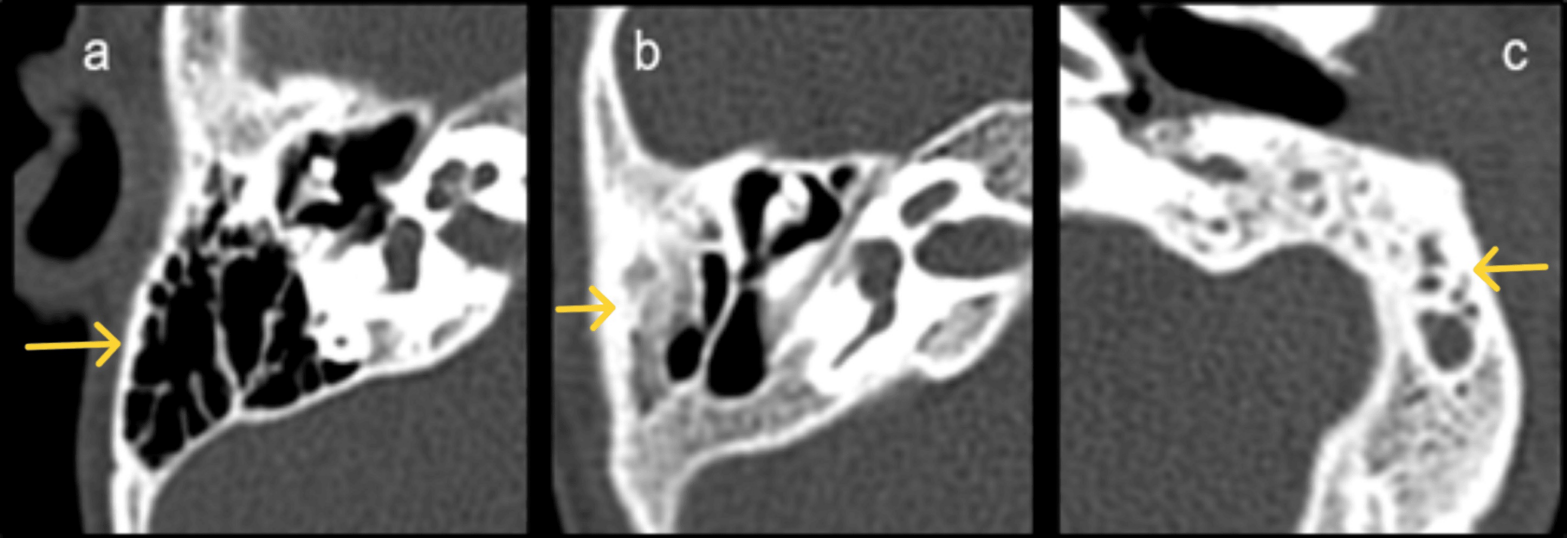
(a)-(c) Axial images of HRCT temporal bone demonstrating mastoid pneumatization pattern. (a) Cellular pattern, (b) diploic pattern, and (c) sclerosed pattern. HRCT: high-resolution computed tomography.

Bilateral normally aerated mastoid cells were observed in 81.25% of patients, while 6.25% demonstrated bilateral mucoinflammatory changes and 3.13% showed diploic or sclerosed patterns (Figure 1). Two patients (6.25%) had unilateral left-sided mucoinflammatory changes. The aditus was aerated bilaterally in 93.75% of cases, with one patient (3.13%) showing bilateral stenosis and another (3.13%) exhibiting a widened left mastoid with collection.

Bilateral aeration of the middle ear cavity was seen in 90.63% of patients. Two cases demonstrated unilateral left-sided and one (3.13%) bilateral mucoinflammatory changes. All patients showed an intact ossicular chain with no detectable abnormalities.

Sigmoid sinus, jugular bulb, and dural variations

The sigmoid sinus was bilaterally normal in 50% of cases, lateral in 37.5%, and anterolateral in 9.3%; one patient (3.1%) had a unilateral right-sided anterolateral sinus. Emissary veins were bilaterally normal in 84.38%, while 9.3% had a unilateral right-sided widened canal, and 6.25% had bilateral widening. The dural plate was bilaterally normal in 53.13%, bilaterally low-lying in 34.38%, and unilaterally low-lying on the left in 12.5%.

**Figure 2:**
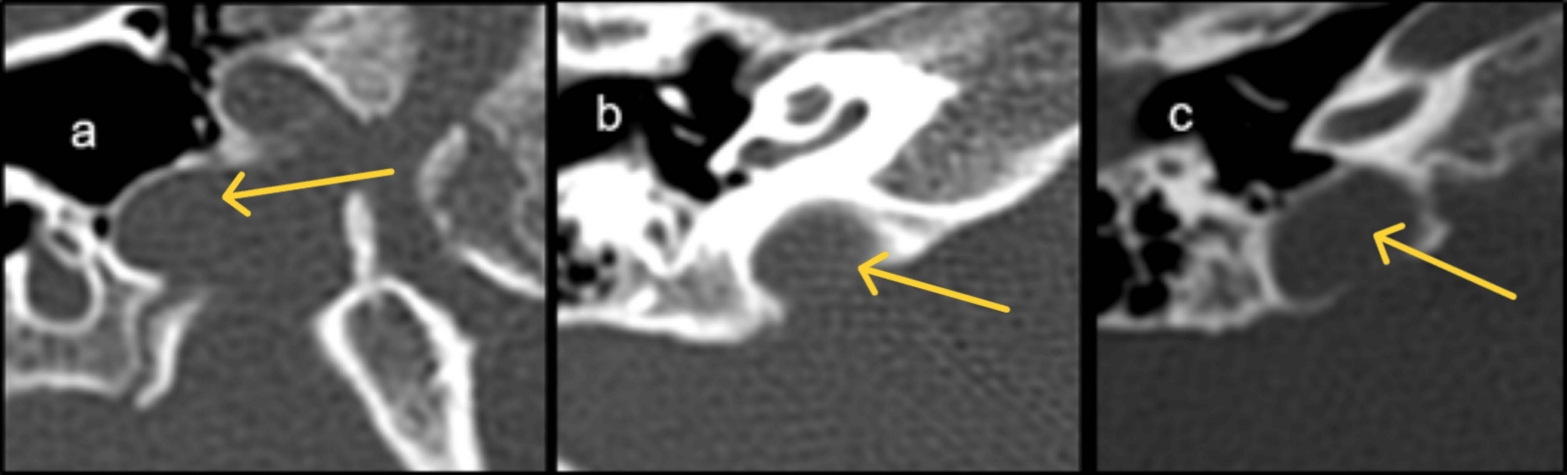
(a)-(c) Axial images of HRCT temporal bone demonstrating the position of the jugular bulb. (a) Normal, (b) high riding, and (c) high-riding with focal dehiscence. HRCT: high-resolution computed tomography.

The jugular bulb was normally positioned in 71.88% of patients, while 18.75% had a high-riding bulb, 6.25% showed bony dehiscence, and one (3.13%) demonstrated unilateral right-sided dehiscence (Figure 2). The cochlear aqueduct was bilaterally normal in 96.8%, with stenosis noted in one case (3.13%).

Vestibular anatomy

A bilaterally normal vestibule was present in 90.63% of patients, with dilation in 6.25% and a small vestibule in 3.13%. The vestibular aqueduct was normal bilaterally in 81.25% of patients, widened in 12.5%, and absent in 6.25%. Bilaterally normal semicircular canals were found in 93.75%, while 6.25% had bilateral anomalies.

Facial nerve and facial recess anatomy

**Figure 3:**
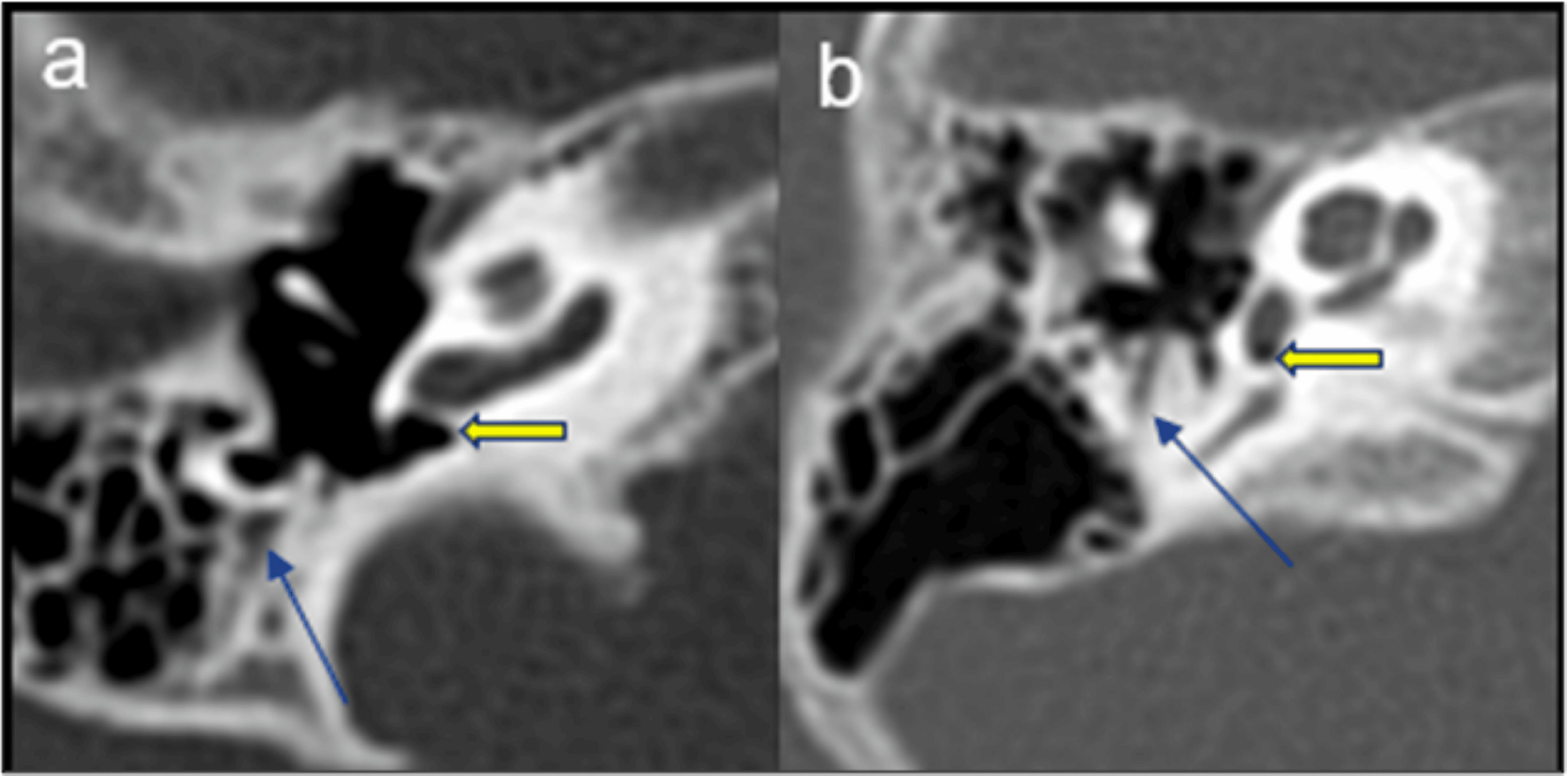
(a) and (b) Axial images of HRCT temporal bone demonstrating descending segment of the facial nerve canal. (a) Normal and (b) overhanging the round window. Blue arrow: descending segment of facial nerve, yellow arrow: round window. HRCT: high-resolution computed tomography.

**Figure 4:**
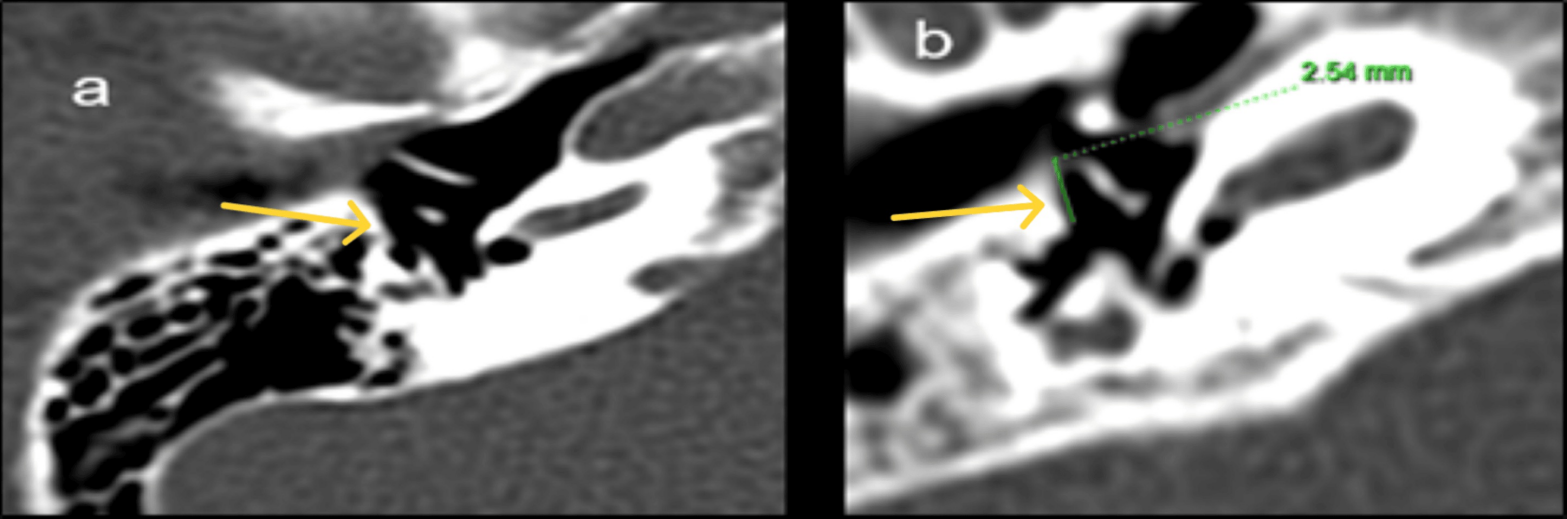
(a) and (b) Axial images of HRCT temporal bone demonstrating facial recess anatomy. (a) Normal and (b) narrow <3 mm. HRCT: high-resolution computed tomography.

Most patients (93.75%) exhibited a normal bilateral facial nerve course. One patient (3.13%) showed bilateral and another (3.13%) unilateral right-sided overhanging of the facial nerve at the round window (RW) (Figure 3). The facial recess (FR) was normal in most cases, though one (3.12%) had bilateral narrow recesses and one (3.12%) unilateral narrow recess on the right side (Figure 4).

Cochlear and related structures

The cochlear aperture was normal bilaterally in 84.38% of patients, widened in 12.5%, and stenosed in 3.12%; three patients exhibited unilateral right-sided widening.

Cochlear duct length (CDL) could not be measured in one patient due to cystic cochleovestibular malformation (incomplete partition (IP) type I). Among the remaining 31 patients, 93.1% had a normal bilateral CDL, one (3.44%) had a short CDL, and one (3.44%) had a long CDL.

The stapes footplate was bilaterally normal in 75% of patients, short in 15.63%, and long in 3.13%; 6.25% showed a unilateral right-sided short variant. The oval window was normal in 81.25%, widened bilaterally in 15.63%, and stenosed unilaterally on the right in 3.13%.

The round window was bilaterally normal in 53.13%, widened bilaterally in 28.13%, and widened unilaterally on the right in 12.5%. Bilateral and unilateral right-sided stenosis were each seen in 3.13% of cases. Among the 19 patients who underwent surgery, the round window (RW) morphology was predominantly C-shaped in 13 patients, followed by triangular in three patients and quadrangular shape in two patients. One patient had an atretic round window and required a promontory cochleostomy. All remaining patients were approached using the extended round window cochleostomy technique.

Inner ear malformations (IEMs) and cochlear nerve findings

**Figure 5:**
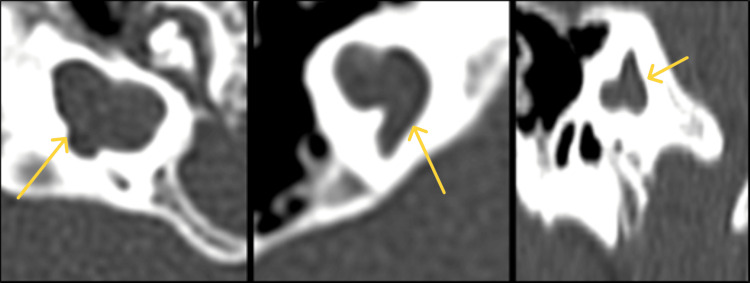
Cystic cochlea-vestibular malformation; type I incomplete partition with cochlear nerve aplasia.

**Figure 6:**
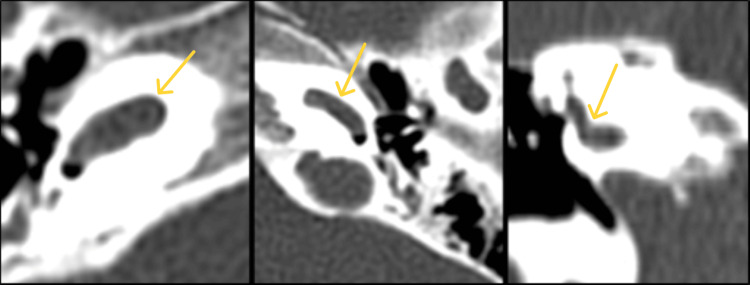
CH II type cochlear hypoplasia with abnormal vestibule, absent superior semicircular canal, and cochlear nerve aplasia. CH: cochlear hypoplasia.

**Figure 7:**
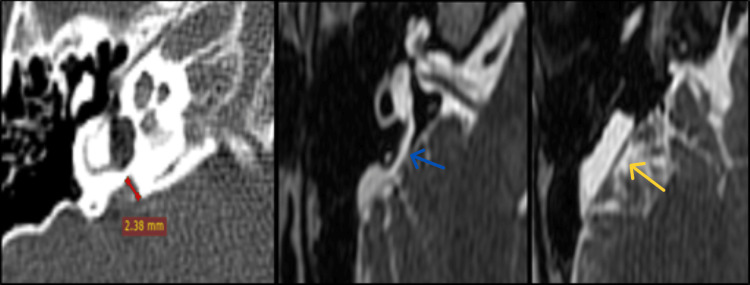
Enlarged vestibular aqueduct (red line) with enlarged endolymphatic duct (blue arrow) and sac (yellow arrow).

**Figure 8:**
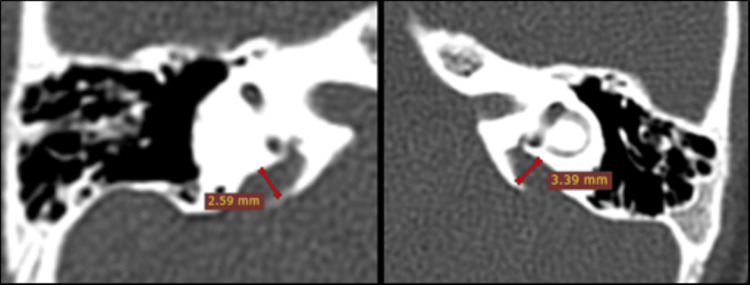
Bilateral enlarged vestibular aqueduct (red lines).

**Figure 9:**
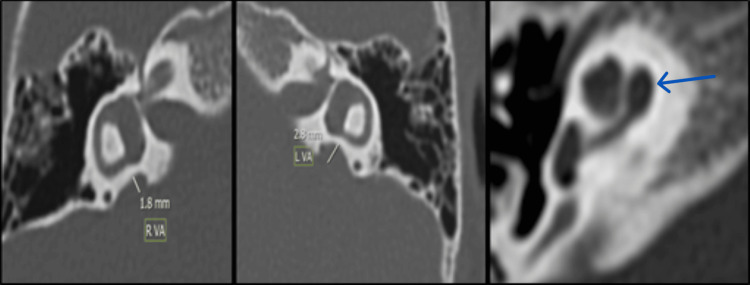
Mondini incomplete partition II deformity; bilateral large vestibular aqueduct (yellow lines), defective modiolus, and wider cochlear canal. Fused apical and middle turn of the cochlea (blue arrow). RVA: right vestibular aqueduct, LVA: left vestibular aqueduct.

Most patients (84.37%) had normal inner ears, while five (15.63%) demonstrated anomalies: one with cystic cochlea-vestibular malformation (IP type I) with absent lateral semicircular canal on both sides (Figure 5). One with cochlear hypoplasia (CH II type), abnormal vestibule, absent semicircular canals, and absent cochlear nerve (Figure 6). Large vestibular aqueduct syndrome (LVAS) with enlarged endolymphatic duct and sac (Figure 7). One with a bilateral enlarged vestibular aqueduct (Figure 8). One with incomplete partition II deformity, bilateral large vestibular aqueduct, defective modiolus, and wider cochlear canal (Figure 9). Five cases of inner ear malformation were detected in our study.

Potential difficulty score (PDS) grading and surgical details

Based on the potential difficulty score (PDS), 90.63% of patients were classified as Grade I, 3.13% as Grade II, 3.13% as Grade III, and one patient (3.13%) as Grade I on the right and Grade II on the left.

Of the 32 patients, 19 (59.37%) underwent surgery, while 13 (40.63%) were not operated. Among the operated patients, 17 (89%) underwent right-sided implantation and two (11%) left-sided. The transmastoid facial recess approach was used in all cases.

Among the five patients with inner ear anomalies, three (60%) underwent surgery and two (40%) were not operated. All three operated anomalous cases experienced intraoperative perilymphatic gushers, consistent with their imaging findings. In 18 operated patients, the extended round window technique was employed, and in one patient, promontory cochleostomy was employed. Intraoperative planning and postoperative recovery were favourable in all cases, with normal postoperative X-rays and no reported surgical and perioperative complications.

## Discussion

Cochlear implantation has revolutionized the treatment of severe to profound sensorineural hearing loss, providing significant auditory and communicative benefits to both children and adults. Advances in cross-sectional imaging--particularly high-resolution computed tomography (HRCT) and magnetic resonance imaging (MRI)--have been pivotal in patient selection, preoperative planning, and postoperative assessment. HRCT offers detailed visualization of the temporal bone, bony labyrinth, and middle ear anatomy, whereas MRI provides superior soft-tissue contrast, allowing evaluation of the membranous labyrinth and the cochleovestibular nerves. Together, these modalities not only identify contraindications but also guide surgical strategy and predict possible complications.

In the present study of 32 patients evaluated for cochlear implantation, cross-sectional imaging findings were systematically analyzed to determine their implications for candidacy, surgical planning, and postoperative outcomes. The majority of patients were aged between one and three years, consistent with global recommendations favoring early implantation for optimal auditory and language development [9]. A mild male predominance was noted, in keeping with prior reports suggesting a slightly higher incidence of congenital hearing loss in males [10].

Inner ear malformations (IEMs)

Inner ear malformations were detected in 15.6% of patients, aligning with the prevalence range of 10-20% reported in previous studies [11,12]. The spectrum of anomalies included cochlear hypoplasia, incomplete partitions (IPs), large vestibular aqueduct syndrome (LVAS), and isolated vestibular or semicircular canal malformations. LVAS was the most frequent anomaly, observed in 60% of patients with IEMs. This is slightly higher than the 20-30% reported by Arcand et al. [13], likely reflecting sample variation and referral bias. All LVAS cases in our series were bilateral, corroborating with the 88% bilateral involvement described by Berrettini et al. [14].

Cochlear hypoplasia was seen in 20% of malformed ears, often associated with internal auditory canal (IAC) narrowing, consistent with Papsin et al. [15], who emphasized the coexistence of IAC anomalies with posterior labyrinthine dysplasias. No cases of complete labyrinthine or cochlear aplasia were encountered, consistent with their reported rarity [11,16]. In two patients, IAC hypoplasia on HRCT corresponded to absent cochlear nerves on MRI, consistent with Glastonbury et al. [17], who reported cochlear nerve aplasia in most cases of IAC narrowing.

Cochlear nerve abnormalities were identified in four ears (40% of IEM cases), higher than the 12-18% prevalence reported by Parry et al. and McClay et al. [18]. This may reflect targeted imaging in patients with suspected cochlear nerve deficiency. The strong correlation between cochlear nerve absence and poor surgical candidacy highlights the indispensable role of MRI in preoperative assessment. Furthermore, perilymphatic gushers were encountered exclusively in patients with IEMs, emphasizing the predictive value of imaging in anticipating intraoperative complications.

Preoperative anatomical assessment

Evaluation of mastoid pneumatization, middle ear structures, and vascular variants is essential in predicting surgical accessibility and risk. In this study, 81.25% of patients exhibited well-aerated mastoids, which is favorable for the transmastoid approach. Mild mucosal inflammatory changes were observed in 12.5%, while 6.25% had sclerotic mastoids, potentially complicating electrode insertion, which were similar findings with Lynch et al. 2019 [19].

Sigmoid sinus variations were frequent, with 37.5% showing lateral or anterolateral positioning, which may increase the risk of sinus injury during posterior tympanotomy as described by Harrison et al. 2017 [20]. A high-riding jugular bulb was noted in 18.75% of cases-an important consideration given its potential to limit surgical exposure and increase intraoperative bleeding risk, which is in sync with Middleton et al. 2020 [21].

Vestibular morphological variations, including vestibular dilation (6.25%) and hypoplasia (3.13%), were also noted. Abnormal vestibular aqueducts were present in 18.75% (12.5% widened and 6.25% absent), both of which may influence vestibular function and increase the likelihood of postoperative imbalance. These findings support prior reports that vestibular anatomy significantly affects the risk of post-implant vertigo or disequilibrium [22,23]. Thus, thorough preoperative vestibular assessment and patient counseling are recommended.

Cochlear aperture and cochlear duct length

The cochlear aperture, or bony cochlear nerve canal (BCNC), represents the conduit through which the cochlear nerve enters the cochlea. Its dimensions are critical for assessing nerve integrity and predicting cochlear implant outcomes. In our study, a normal cochlear aperture was identified in 84.38% of patients, a stenosed aperture in 3.12 %, and a widened aperture in 12.5%. Stenosis correlated strongly with cochlear nerve hypoplasia or aplasia, indicating potential non-candidacy for implantation and the need to consider alternatives such as auditory brainstem implantation. Conversely, widened apertures were frequently associated with LVAS and other IEMs, often predisposing to intraoperative perilymphatic gushers and postoperative vestibular symptoms. Preoperative recognition of these findings enables the surgeon to anticipate potential challenges and plan the surgical approach accordingly.

**Figure 10:**
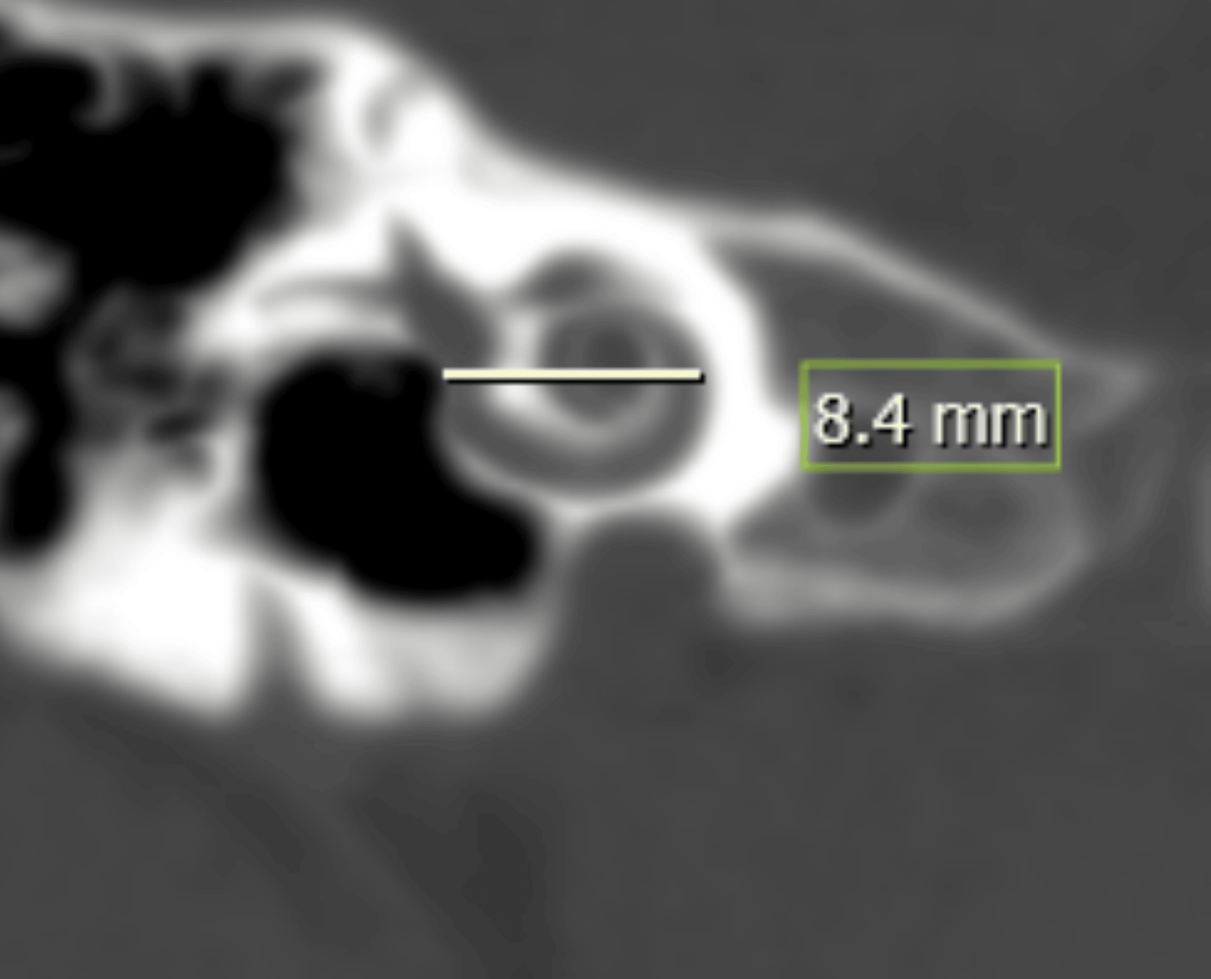
Cochlear duct length (CDL) measurement. A-value = distance from the round window through the modiolus to the opposite lateral wall on mid-modiolar CT (white line).

Cochlear duct length (CDL) is a critical determinant for electrode selection and full cochlear coverage (Figure 10). Using Jolly's and Escudé's formulas (CDL = 4.16 × A − 3.98/2.7), CDL could not be estimated in one patient with incomplete partition type I due to cystic morphology, reflecting the limitation of linear formulas in malformed cochleae. Among the remaining 31 patients, 93% had CDL values within the normal range (28-30 mm). Advances such as three-dimensional reconstruction, MRI-based segmentation, and advanced software provide more accurate CDL estimations, particularly in anomalous cochleae [24,25].

Oval and round window anatomy

The stapes footplate appeared normal in 75% of cases, with mild morphological variations in the remainder. The oval window was normal in 81.25% of patients; anatomical variations in others could affect access or increase the risk of fluid leak. While otosclerosis was not detected, its recognition remains important since far-advanced otosclerosis can pose surgical challenges due to ossification and increased risk of perilymphatic gusher, though implantation remains effective [26].

Round window (RW) visualization through the facial recess (FR) is fundamental to atraumatic electrode placement into the scala tympani. In this cohort, 53% had normal RWs, 28% showed widening, and 6% narrowing. Narrow RWs may obscure visualization, necessitating extended approaches or cochleostomy. The FR width, normally >3 mm, was narrowed in 6% of patients--an important predictor of surgical difficulty [27,28]. High-resolution imaging of the FR, RW niche, and facial nerve course, therefore, plays a vital role in determining the feasibility of the transmastoid facial recess approach [29].

Surgical and postoperative outcomes

Favorable outcomes were achieved in 19 of 32 patients who underwent surgery. The transmastoid facial recess approach was used in all cases; two patients required left-sided implantation due to an anomalous right facial nerve course overhanging the round window. Intraoperative perilymph gushers occurred in five patients with IEMs, consistent with reports linking such complications to bony defects and abnormal cochlear anatomy. Despite these challenges, all 19 patients demonstrated improved surgical preparedness and normal imaging findings.

The consistent application of the extended round window technique across all surgeries highlights its utility in ensuring safe electrode insertion and preserving cochlear structures. Importantly, preoperative imaging accurately predicted the need for modified surgical approaches in all complex cases, underscoring its predictive value.

## Conclusions

In conclusion, this study reinforces the vital contribution of CT and MRI to the pre-surgical workup for cochlear implantation. By identifying anatomical anomalies, inner ear malformations, and other important variations, imaging provides essential guidance in tailoring the surgical approach to minimize complications and optimize surgical and perioperative outcomes. The complementary use of both CT and MRI is indispensable for a comprehensive understanding of each patient’s unique anatomy, ultimately leading to improved safety and efficacy in cochlear implantation surgeries. As demonstrated in this study, detailed imaging not only aids in patient selection but also helps predict the surgical course and intraoperative results, making it an essential component of the pre-operative planning process for cochlear implantation.
